# Caught on CT! The Case of the Hemodynamically Stable Ruptured Abdominal Aortic Aneurysm

**DOI:** 10.21980/J8B07B

**Published:** 2020-07-15

**Authors:** Matthew Patrick Weber, Megan Stobart-Gallagher

**Affiliations:** * Thomas Jefferson University Hospitals, Department of Emergency Medicine, Philadelphia, PA

## Abstract

Rupture of an abdominal aortic aneurysm (AAA) is a surgical emergency with significant associated mortality. This case report discusses a 70-year-old woman presenting to the emergency department (ED) with vague and progressive abdominal pain and relatively benign vital signs and physical exam. She was ultimately diagnosed with a ruptured infrarenal 6.8cm abdominal aortic aneurysm seen on computerized tomography (CT) imaging. Found to be stable, she was transferred to an affiliate tertiary care facility and underwent endovascular repair of the aneurysm. The surgery was successful, and she has since recovered nicely. As reported at her most recent follow up appointments. A timely diagnosis of a ruptured AAA is vital for any chance of a successful outcome. However, in patients with normal vital signs and an unremarkable physical exam, this diagnosis can be incredibly difficult. As such, the need for a broad differential in the setting of abdominal pain is imperative to avoid missing the diagnosis. The use of point of care ultrasound (POCUS) can further aid in this process by providing a quick and affordable screening option to rule out potential aneurysms.

**Topics:**

Abdominal Aortic Aneurysm, Ruptured Abdominal Aortic Aneurysm, CT imaging, Tangential Calcium Sign, Point of Care Ultrasound.

**Figure f1-jetem-5-3-v14:**
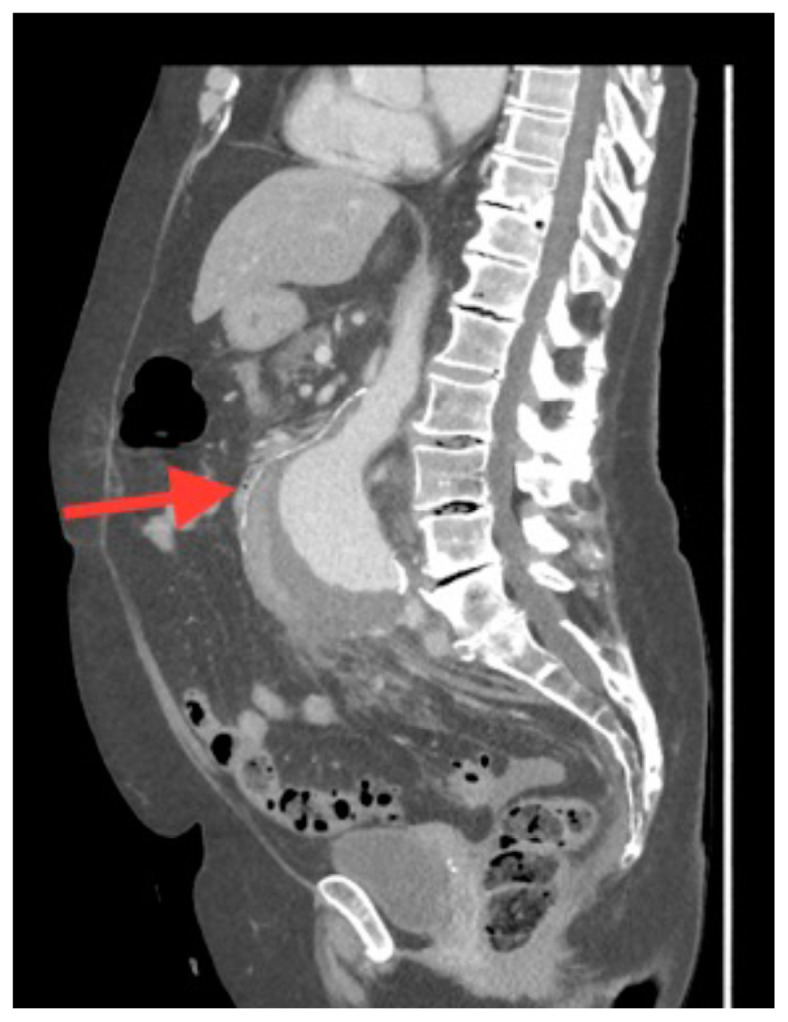


**Figure f2-jetem-5-3-v14:**
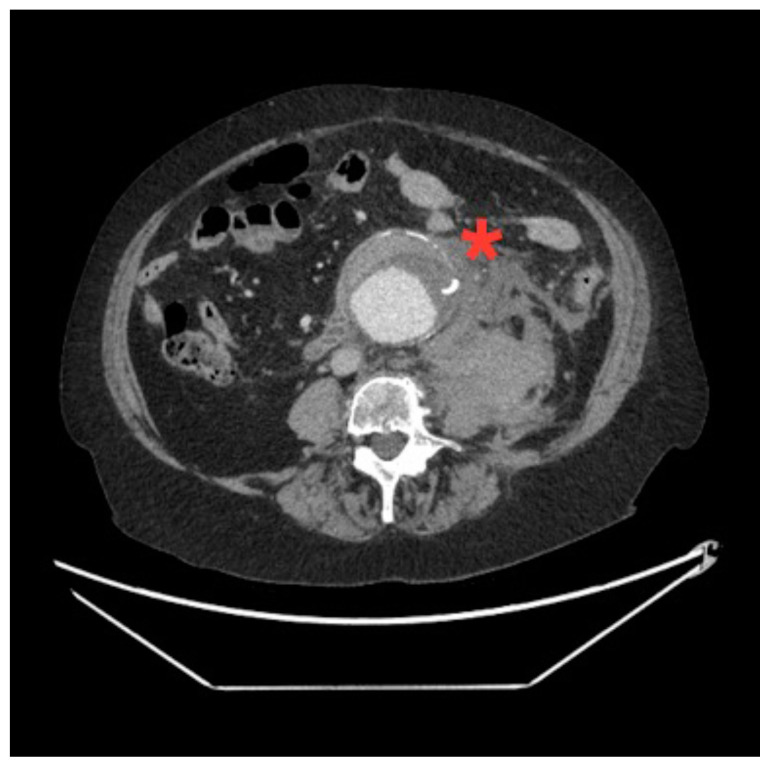


**Figure f3-jetem-5-3-v14:**
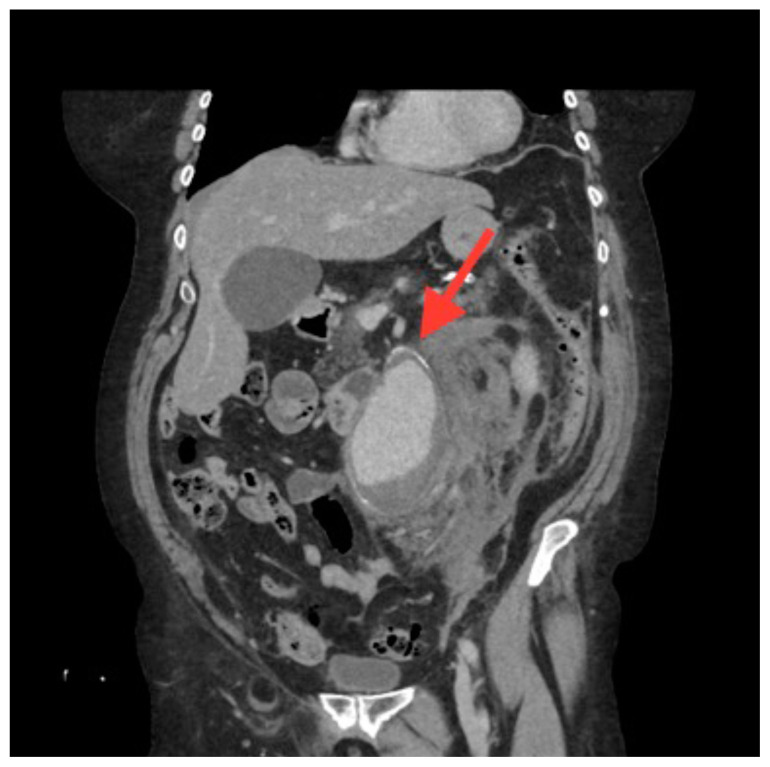


## Presenting concerns and clinical findings

[Fig f1-jetem-5-3-v14][Fig f2-jetem-5-3-v14][Fig f3-jetem-5-3-v14]A 70-year-old female presented to the emergency department with a two-week history of vague abdominal pain worsening over the past few days. Patient noted the pain was primarily in the lower abdomen, and it was associated with nausea, non-bilious and non-bloody vomiting, and constipation. Review of systems was positive for fatigue, but devoid of back pain, urinary or vaginal symptoms. She had a past medical history of hypertension and asthma, with a remote history of cigarette smoking. At presentation the patient was afebrile with a heart rate of 108 bpm, a blood pressure of 108/58, a respiratory rate of 18 bpm and an oxygen saturation of 96% on room air. On physical exam, she was uncomfortable appearing with diaphoresis. Her abdomen was soft with tenderness across the entire lower abdomen without rebound, guarding, distension, pulsatile masses, or hernias. Notable laboratory studies included a sodium of 130 meq/L, a blood urea nitrogen of 24 mg/dL, a creatinine of 1.4 mg/dL, a white blood cell count of 15.5 × 109 cells per liter, and a hemoglobin of 8.7 g/dL. A CT of the abdomen and pelvis with intravenous contrast was obtained providing the diagnosis.

## Patient Course

The diagnostic assessment of this patient’s presentation was challenging. Her mild tachycardia and relative hypotension did not depict an individual with hemodynamic instability expected in the case of a ruptured AAA. Once the diagnosis was made, vascular surgery at an affiliate tertiary care center was consulted, and the patient was immediately transferred. The patient underwent emergent endovascular repair of her ruptured aneurysm and was discharged a few days later. At her one-month follow-up, she was asymptomatic and progressing well.

## Significant findings

The associated images demonstrate the transverse, sagittal, and coronal views of a 6.8 cm infrarenal ruptured AAA continuous with a 4 cm right common iliac aneurysm (transverse, sagittal and coronal). Active hemorrhage was seen contained within the aortic wall, and retroperitoneal bleeding can be appreciated with asymmetric enlargement of the left psoas muscle (coronal - red arrow).^1^ Plaque and calcifications with a residual opacified true lumen is also present (transverse – red star, sagittal – red arrow). Known as the tangential calcium sign, this is a common radiologic finding of AAAs.^2^

## Discussion

Risk of developing an AAA has been increasing in recent years with a documented prevalence of 4 to 7 percent of the general population.^3^ Male sex, hypertension, and hyperlipidemia are associated with an increased risk of aneurysm development. A history of smoking is the greatest predictor for AAA formation.^4^,^5^ Outpatient screening for AAA formation is historically limited to men between 65–75 years of age with a smoking history.^6^ Once an aneurysm is identified, treatment focuses on the prevention of further growth by managing the underlying cardiovascular pathophysiology with the ultimate goal of preventing rupture.

Rupture is defined as “the presence of blood outside adventitia of the dilated aortic wall.” This is a rather heterogeneous event that can result in profuse retroperitoneal hemorrhage or may be more limited by an anatomic tamponade.^7^ The degree of bleeding will dictate the patient’s presentation; however, a ‘clinical triad ‘ of abdominal pain, hypotension and a pulsatile mass is classically associated with the presence of pathology.^8^ However, this classic triad is not often seen on presentation, as highlighted by our patient. A majority of patients with ruptured AAA report some degree of pain, but few patients present with the expected hypotension and tachycardia.^9^ Atypical presentations exacerbate a difficulty in diagnosis because clinical suspicion may not be sufficient to order imaging needed to make the diagnosis.

The role of point of care ultrasound may help fill this natural diagnostic gap. Because clinicians must maintain a broad differential diagnosis in the setting of abdominal or back pain, POCUS could provide a cheap and efficient method to rule out cannot miss diagnoses. Nixon et.al have reported diagnosing AAA using POCUS with the 100% sensitivity and 93% specificity. 10 Although a timely diagnosis was made in this case, the use of POCUS could have further expedited the diagnostic process and served as a cost-effective screening tool.

In conclusion, this case highlights the importance of a thorough history and high suspicion in the setting of a patient with risk factors for AAA presenting with abdominal pain. Although traditionally clinicians may have been taught to expect marked hypotension or hemodynamic instability in patients with ruptured AAA, relative hypotension or normal blood pressures are fairly common at presentation. Therefore, clinicians must maintain a broad differential in this setting. Now and in the future, POCUS should be a major tool for clinicians in this challenging decision-making process to assure such a devastating diagnosis will not be missed.

## Supplementary Information














