# Supplementary figures and images for: Caught on CT! The Case of the Hemodynamically Stable Ruptured Abdominal Aortic Aneurysm
Source: J Educ Teach Emerg Med. 2020 Jul 15;5(3):V14–7. doi: 10.21980/J8B07B (PMC10332550; doi:10.21980/J8B07B)

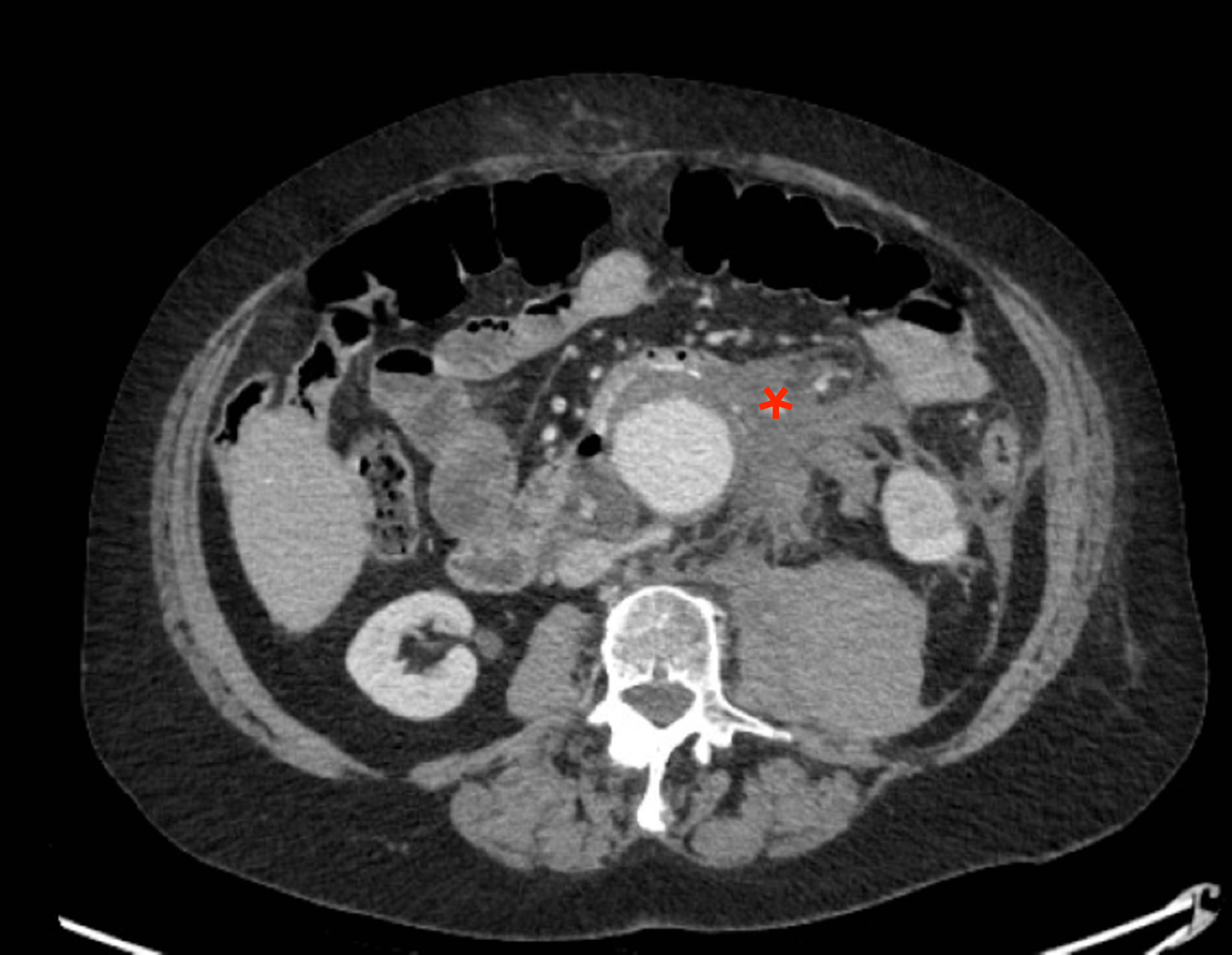

Supplement: Supplementary file 1 [file jetem-5-3-v14-supp1.jpg]

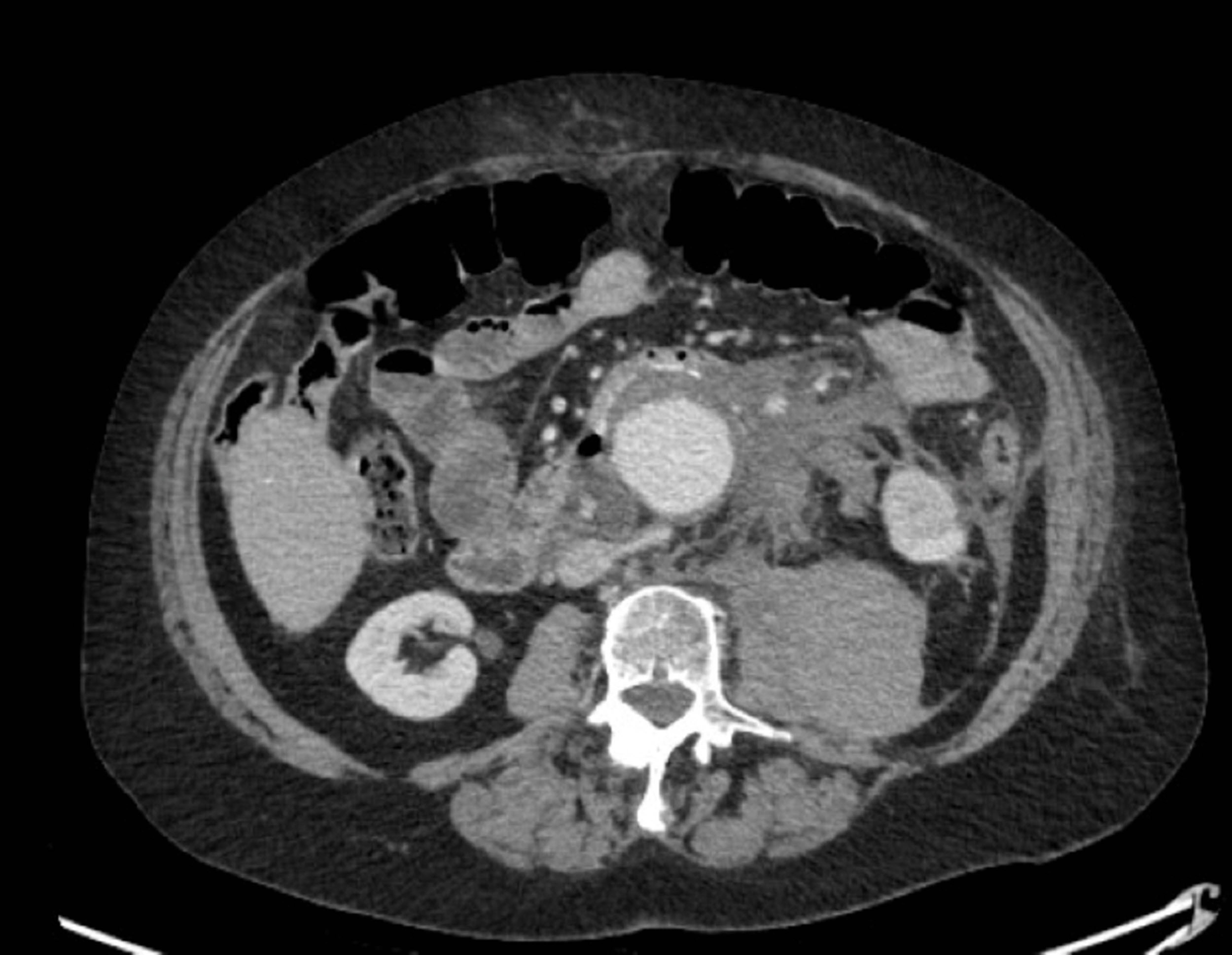

Supplement: Supplementary file 2 [file jetem-5-3-v14-supp2.jpg]

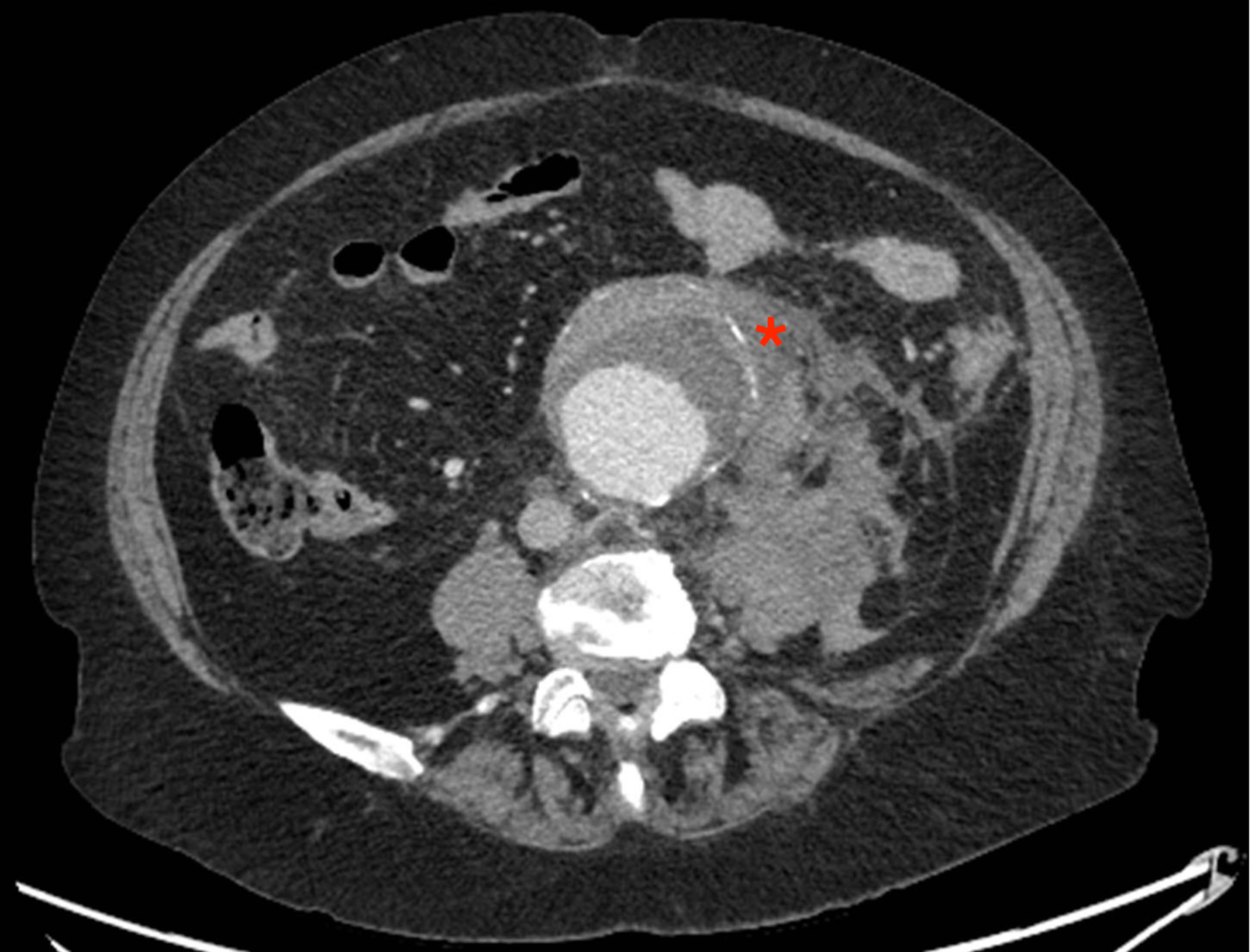

Supplement: Supplementary file 3 [file jetem-5-3-v14-supp3.jpg]

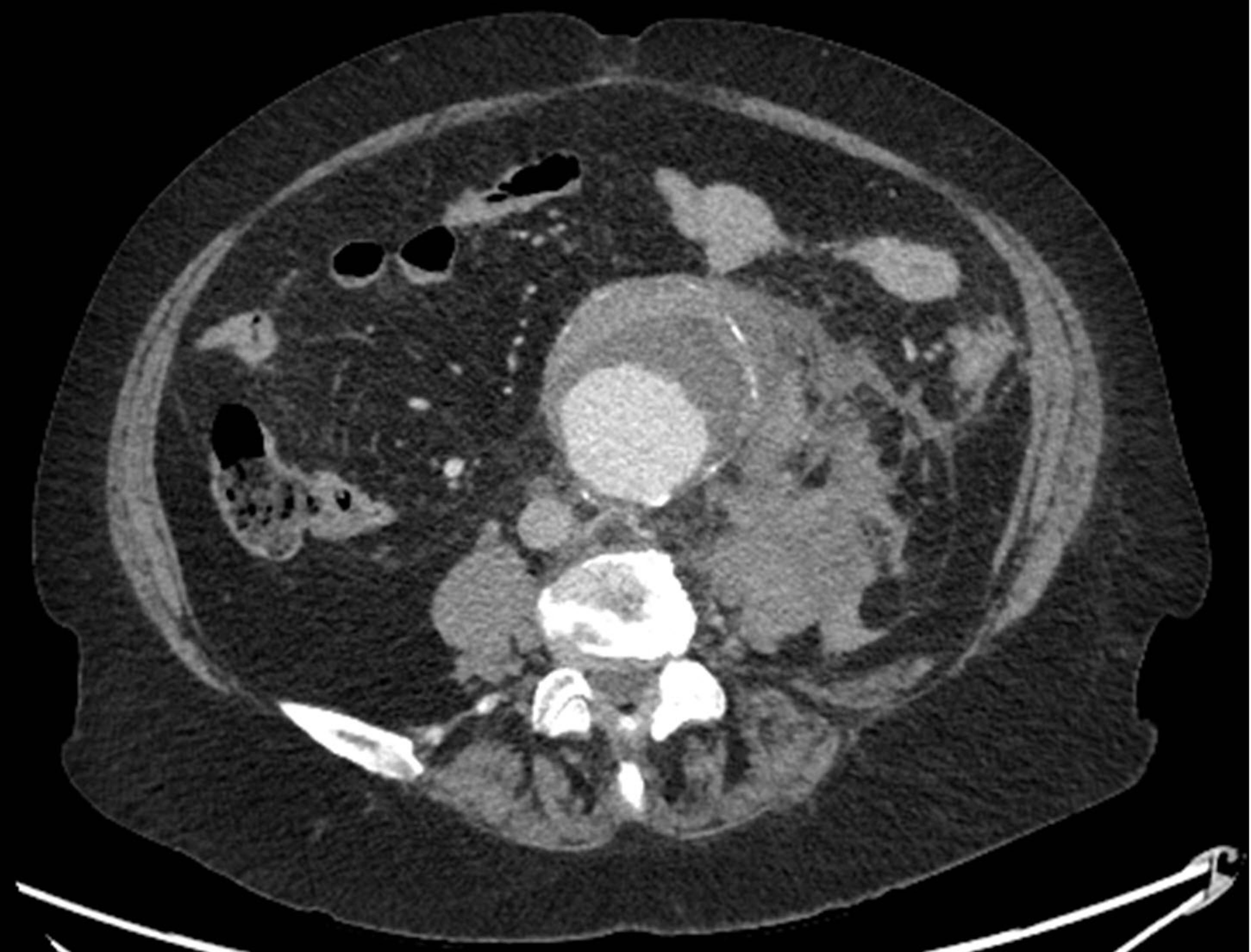

Supplement: Supplementary file 4 [file jetem-5-3-v14-supp4.jpg]

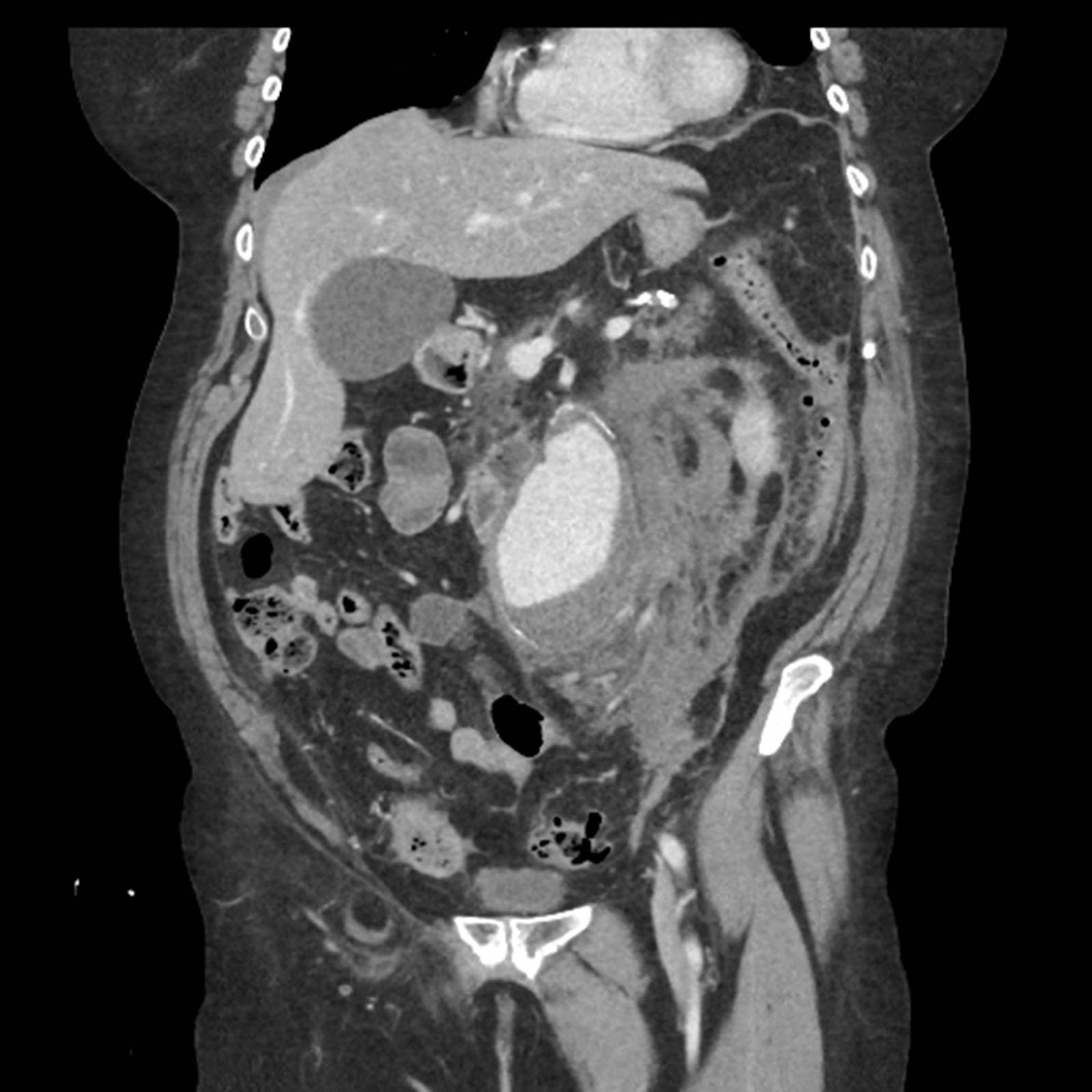

Supplement: Supplementary file 5 [file jetem-5-3-v14-supp5.jpg]

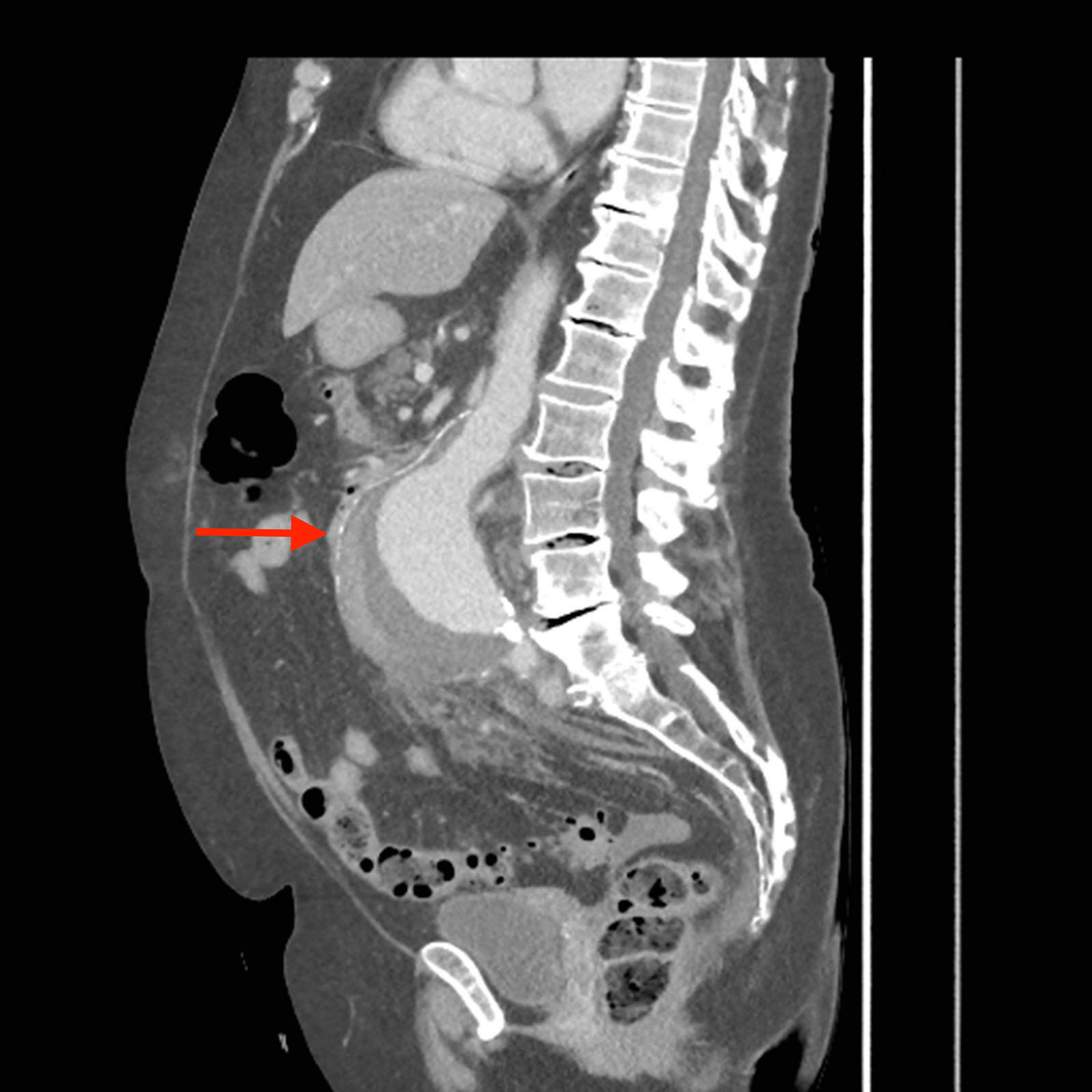

Supplement: Supplementary file 6 [file jetem-5-3-v14-supp6.jpg]

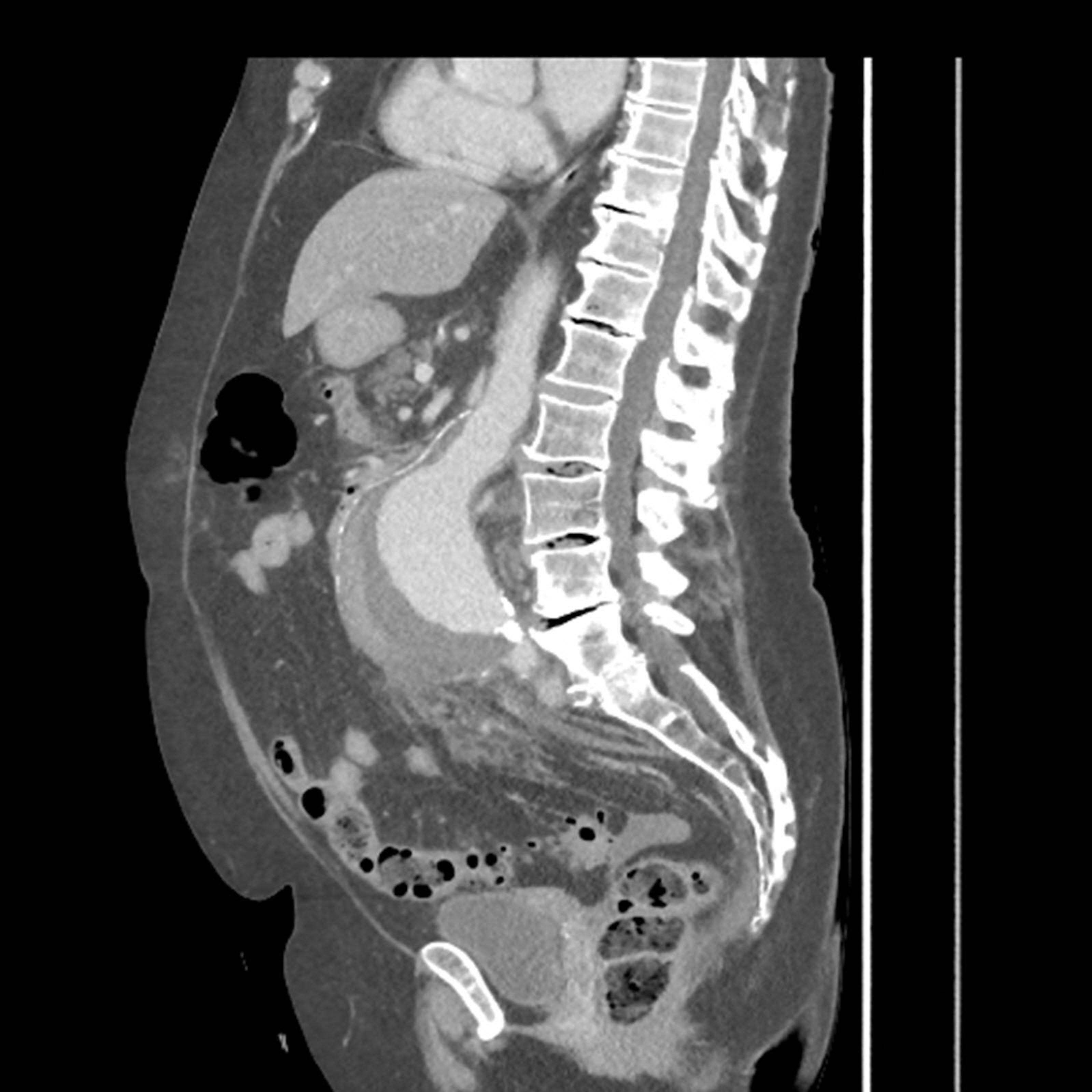

Supplement: Supplementary file 7 [file jetem-5-3-v14-supp7.jpg]
